# Subtype-Consistent Upregulation of Ferroptosis-Associated Pathways in Breast Cancer with Heterogeneous Prognostic Implications and Systemic Response to Cryoablation

**DOI:** 10.3390/ijms27083446

**Published:** 2026-04-12

**Authors:** Kacper Boroń, Agata Panfil, Tomasz Sirek, Agata Sirek, Nikola Zmarzły, Michalina Wróbel, Zbigniew Wróbel, Dariusz Boron, Piotr Ossowski, Martyna Stefaniak, Paweł Ordon, Grzegorz Wyrobiec, Piotr Wyrobiec, Wojciech Kulej, Natalia Lekston, Beniamin Oskar Grabarek

**Affiliations:** 1 Department of Plastic Surgery, Faculty of Medicine, Academia of Silesia, 40-555 Katowice, Poland; drtstierka@gmail.com (T.S.); agatasirek06@gmail.com (A.S.); 2 Faculty of Medicine and Health Sciences, Andrzej Frycz Modrzewski University in Kraków, 30-705 Kraków, Poland; dariusz@boron.pl; 3 Department of Gynecology and Obstetrics, TOMMED Specjalisci od Zdrowia, 40-055 Katowice, Poland; 4 Department of Gynecology and Obstetrics with Gynecologic Oncology, Ludwik Rydygier Memorial Specialized Hospital, 31-826 Kraków, Poland; 5 Collegium Medicum, WSB University, 41-300 Dabrowa Gornicza, Poland; agatapanfil875@gmail.com (A.P.); nikola.zmarzly@gmail.com (N.Z.); wrobelmichalina@o2.pl (M.W.); zbi.wrobel@gmail.com (Z.W.); drpiotrossowski15@gmail.com (P.O.); martynastefaniakk@gmail.com (M.S.); pawelordon4@gmail.com (P.O.); wojciechkulej87@gmail.com (W.K.); lekstonnatalia@gmail.com (N.L.); 6 Department of Plastic and Reconstructive Surgery, Hospital for Minimally Invasive and Reconstructive Surgery in Bielsko-Biała, 43-316 Bielsko-Biala, Poland; 7 Medi-Lab SC, Wróbel i Wspólnicy, 58-100 Świdnica, Poland; 8 Department of Histology and Cell Pathology in Zabrze, Faculty of Medical Sciences in Zabrze, Medical University of Silesia in Katowice, 40-055 Katowice, Poland; gwyrobiec@sum.edu.pl (G.W.); piotruswyr@interia.pl (P.W.)

**Keywords:** breast neoplasms, ferroptosis, gene expression regulation, neoplastic, oxidative stress, biomarkers, tumor

## Abstract

Ferroptosis is an iron-dependent form of regulated cell death driven by lipid peroxidation and oxidative stress, increasingly implicated in cancer biology. However, its molecular regulation across breast cancer subtypes and its potential systemic manifestations remain incompletely understood. The aim of this study was to identify ferroptosis-associated molecular alterations that are largely shared across subtypes and to evaluate their systemic reflection following localized tissue injury. Tumor and matched normal breast tissues representing major molecular subtypes were analyzed. Global mRNA and miRNA expression profiling was performed using microarrays, followed by validation of selected genes using quantitative reverse transcription polymerase chain reaction (qRT-PCR) and enzyme-linked immunosorbent assay (ELISA). Functional enrichment and protein–protein interaction analyses were conducted to characterize associated pathways. In addition, systemic responses were assessed in patients undergoing fibroadenoma cryoablation through longitudinal blood sampling. Six ferroptosis-related genes (*SLC7A11*, *GPX4*, *FTH1*, *NQO1*, *NFE2L2*, *SQSTM1*) demonstrated consistent upregulation across all breast cancer subtypes, with higher expression observed in more aggressive tumors. These genes are functionally linked to antioxidant defense, iron metabolism, and oxidative stress regulation, and their coordinated expression pattern is consistent with activation of NRF2-dependent cytoprotective pathways. Downregulation of selected miRNAs may contribute to this expression profile but likely represents a secondary regulatory mechanism. Survival analysis revealed heterogeneous and subtype-dependent associations, with limited and gene-specific prognostic relevance. Cryoablation induced transient increases in circulating levels of the analyzed proteins, reflecting systemic responses to localized tissue injury. In conclusion, breast cancer is characterized by a largely shared ferroptosis-associated molecular signature across subtypes; however, its clinical impact appears to be variable and context-dependent. Systemic detection of related molecular signals suggests potential utility as indicators of tissue stress responses, although their role as specific biomarkers of ferroptosis requires further validation.

## 1. Introduction

Breast cancer remains the most frequently diagnosed malignancy among women worldwide and represents a major cause of cancer-related morbidity and mortality despite significant advances in early detection and systemic therapy [1,2]. The disease is biologically heterogeneous and encompasses multiple molecular subtypes—luminal A, luminal B, HER2-positive, and triple-negative breast cancer (TNBC)—each characterized by distinct genetic landscapes, clinical behavior, and therapeutic vulnerabilities [3,4,5]. While subtype-driven treatment strategies have improved outcomes, resistance to therapy and disease recurrence continue to pose substantial clinical challenges, underscoring the need for deeper insight into tumor cell death mechanisms that may be exploited for therapeutic benefit [6,7].

Regulated forms of cell death play a pivotal role in tumor development, progression, and treatment response [8]. Among these, ferroptosis has emerged as a distinct, iron-dependent mode of regulated cell death characterized by lipid peroxidation, redox imbalance, and failure of antioxidant defense systems [9,10]. Unlike apoptosis or necroptosis, ferroptosis is driven by the accumulation of reactive oxygen species within polyunsaturated fatty acid–containing phospholipids, a process tightly regulated by iron metabolism, glutathione availability, and the activity of lipid repair enzymes [11,12,13]. Increasing evidence suggests that ferroptosis is intricately linked to cancer metabolism, tumor aggressiveness, and sensitivity to chemotherapy, radiotherapy, and targeted agents [14,15].

In breast cancer, dysregulation of iron homeostasis, oxidative stress pathways, and lipid metabolism has been reported across molecular subtypes, suggesting a potential role for ferroptosis in tumor biology [16,17]. Experimental studies have demonstrated that induction of ferroptosis may suppress tumor growth, particularly in therapy-resistant and triple-negative tumors, which lack effective targeted treatment options [18,19,20,21]. Conversely, ferroptosis resistance mechanisms may contribute to tumor survival and disease progression [22]. Despite growing interest, the molecular regulation of ferroptosis in human breast cancer tissue—especially in the context of different molecular subtypes—remains incompletely characterized [23,24].

MicroRNAs (miRNAs) represent an additional regulatory layer influencing ferroptosis-related pathways [25]. These small non-coding RNAs modulate gene expression post-transcriptionally and have been implicated in the control of iron metabolism, antioxidant defense, lipid peroxidation, and stress-response signaling [26,27]. Aberrant miRNA expression is a hallmark of breast cancer and varies significantly among molecular subtypes [28]. However, systematic analyses integrating ferroptosis-related mRNA expression with miRNA regulatory networks in clinical breast cancer specimens are still limited [29].

Beyond tumor tissue, increasing attention has been directed toward the systemic molecular consequences of localized tissue injury and ablation [30,31]. Cryoablation of benign breast lesions, such as fibroadenomas, induces acute cellular stress, inflammation, and regulated cell death, offering a unique in vivo model to study early circulating molecular responses associated with specific cell death pathways [32,33]. Evaluating whether ferroptosis-related molecular signatures detected in tumor tissue are reflected in peripheral blood following cryoablation may provide insight into the systemic footprint of ferroptotic signaling and its potential utility as a minimally invasive biomarker [34,35].

Therefore, the aim of the present study was to perform a comprehensive, multilevel characterization of ferroptosis-associated molecular alterations in breast cancer across five major molecular subtypes. By integrating genome-wide mRNA and miRNA microarray profiling with targeted validation at both the transcript and protein levels, we sought to identify both shared and subtype-specific patterns of ferroptosis-related regulation. In addition, by analyzing peripheral blood samples from patients undergoing fibroadenoma cryoablation, we aimed to determine whether early systemic molecular responses reflect key pathways associated with ferroptosis-related stress and redox regulation observed in breast cancer tissue. Through this integrative approach, the study seeks to provide a more nuanced understanding of ferroptosis-associated signaling in breast cancer biology and to explore its potential relevance in the context of biomarker discovery and therapeutic targeting.

## 2. Results

### 2.1. Differential Expression of Ferroptosis-Related Genes in Breast Cancer Subtypes

From the predefined panel of 18 ferroptosis-related mRNAs selected from the MSigDB database, statistical analysis identified significant differential expression for a subset of genes when breast cancer tissue was compared with matched non-neoplastic controls. Analysis of variance (ANOVA) revealed that six mRNAs met the significance threshold (*p* < 0.05) across all breast cancer molecular subtypes, indicating a subtype-independent expression pattern. The remaining genes showed either subtype-restricted significance or did not reach statistical significance in any subtype (Table 1).

Among the universally altered expression, *SLC7A11*, *GPX4*, *FTH1*, *NQO1*, *NFE2L2*, and *SQSTM1* were consistently differentially expressed in luminal A, luminal B HER2−, luminal B HER2+, non-luminal HER2+, and triple-negative breast cancer (TNBC) samples relative to control tissue. These genes demonstrated concordant directionality of change across subtypes, with statistically significant differences maintained after post hoc testing.

In contrast, *HMOX1* exhibited significant differential expression in luminal B HER2−, luminal B HER2+, non-luminal HER2+, and TNBC tumors, but did not reach statistical significance in the luminal A subtype. *AIFM2* (*FSP1*) showed significant alteration in luminal B HER2+, non-luminal HER2+, and TNBC samples, while expression changes in luminal A and luminal B HER2− tumors were not statistically significant.

Several genes displayed subtype-specific significance. *DHCR7* and *SC5D* were significantly altered in luminal A and TNBC samples only, whereas *ACLY* and *SLC25A1* reached statistical significance in luminal B HER2− and TNBC tumors. *HDAC3* showed significant differential expression exclusively in luminal B HER2− samples, while *KAT2B* reached significance only in luminal A tumors. Similarly, *TMEM164* and *SLC39A7* were significantly altered in luminal B HER2+ cancers, whereas *ADGRG1* and *NINJ1* demonstrated significant expression changes solely in non-luminal HER2+ tumors.

No statistically significant differences were observed for the remaining genes outside these subtype-specific contexts. Collectively, these results indicate that, among the selected ferroptosis-related gene set, only a limited subset exhibits consistent differential expression across all breast cancer subtypes, while the majority demonstrate molecular subtype-dependent expression patterns.

### 2.2. Validation of Ferroptosis-Related Gene Expression by RT-qPCR

**Figure 1:**
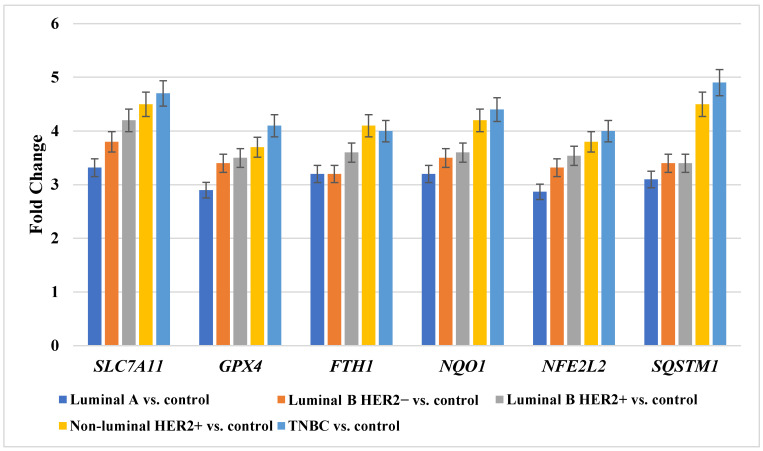
Guantitative reverse transcription polymerase chain reaction (qRT-PCR) confirmation of differential expression of six ferroptosis-related mRNAs across breast cancer molecular subtypes. Data are presented as mean ± SD. *SLC7A11*, Solute carrier family 7 member 11 (cystine/glutamate antiporter, system Xc^−^ light chain); *GPX4*, Glutathione peroxidase 4; *FTH1*, Ferritin heavy chain 1; *NQO1*, NAD(P)H quinone dehydrogenase 1; *NFE2L2*, Nuclear factor erythroid 2-related factor 2; *SQSTM1*, Sequestosome 1; HER2, human epidermal growth factor receptor 2; TNBC, triple-negative breast cancer.

Quantitative reverse transcription PCR (RT-qPCR) was conducted to validate microarray-derived expression patterns of six ferroptosis-related genes (*SLC7A11*, *GPX4*, *FTH1*, *NQO1*, *NFE2L2*, and *SQSTM1*) that were identified as subtype-independent. RT-qPCR analysis confirmed the direction and magnitude of expression changes observed in the microarray dataset across all breast cancer molecular subtypes when compared with matched control tissue. All six genes demonstrated statistically significant upregulation in tumor samples relative to controls (*p* < 0.05), with consistent expression trends across luminal A, luminal B HER2−, luminal B HER2+, non-luminal HER2+, and TNBC subgroups. The highest relative expression levels were generally observed in non-luminal HER2+ and TNBC samples, in agreement with microarray findings. Overall, RT-qPCR results corroborated the robustness and reproducibility of the microarray analysis, supporting the reliability of these genes as consistently altered ferroptosis-related genes in breast cancer (Figure 1).

### 2.3. Differential Expression of miRNAs Associated with Ferroptosis-Related mRNAs Across Breast Cancer Subtypes

Analysis of miRNA expression profiles identified differential regulation of several miRNAs predicted to target ferroptosis-related mRNAs across breast cancer molecular subtypes compared with control tissue. All comparison miRNAs exhibited negative log_2_ fold-change values in tumor samples, indicating reduced expression relative to non-neoplastic controls. The magnitude of downregulation varied among breast cancer subtypes and between individual miRNAs (Table 2).

For miRNAs targeting *SLC7A11*, both hsa-miR-1297 and hsa-miR-26a-5p showed progressive decreases in expression from luminal A to TNBC samples, with the lowest log_2_FC values observed in non-luminal HER2+ and TNBC samples. A similar trend was observed for hsa-miR-18b-3p, predicted to regulate *NQO1*, which demonstrated increasing downregulation across more aggressive subtypes.

MiRNAs associated with *NFE2L2*, including hsa-miR-28-5p and hsa-miR-30a-3p, also displayed reduced expression across all subtypes, with log_2_FC values becoming more negative in HER2-positive and TNBC samples compared with luminal tumors. Overall, the miRNA expression data revealed consistent subtype-dependent differences in the degree of downregulation, while maintaining concordant directionality across all breast cancer subtypes.

### 2.4. Enzyme-Linked Immunosorbent Assay (ELISA)-Based Quantification of Ferroptosis-Related Proteins Across Breast Cancer Subtype

ELISA analysis demonstrated progressively higher concentrations of all analyzed ferroptosis-related proteins in breast cancer tissue compared with control samples, with the highest mean levels observed in non-luminal HER2-positive and triple-negative tumors (Table 3).

### 2.5. Temporal Changes in mRNA and Protein Expression Following Cryoablation in Women with Fibroadenoma

RT-qPCR analysis demonstrated time-dependent changes in the expression of all analyzed mRNAs following fibroadenoma cryoablation (Table 4). For each transcript, fold-change values increased relative to baseline at early post-procedural time points. Statistically significant elevations were consistently observed at 8–12 h (T2) and remained significant at 48–72 h (T3) for all genes. At later time points, including 7 days (T4), 1 month (T5), and 3 months (T6), mRNA expression levels progressively declined and approached baseline values, with no statistically significant differences detected relative to T0.

Serum protein measurements revealed temporal changes in the circulating levels of proteins corresponding to ferroptosis-related genes (Table 5). For all analyzed proteins, concentrations increased significantly at early post-procedural time points, with statistically significant differences observed at 30–60 min (T1), peaking at 8–12 h (T2), and remaining elevated at 48–72 h (T3). For several proteins, significantly increased concentrations were also detected at 7 days (T4). At later follow-up points (T5 and T6), protein levels decreased and stabilized near baseline values, with no statistically significant differences observed compared with pre-procedural measurements.

### 2.6. Functional Enrichment and PPI Analysis of Core Ferroptosis-Related Genes

**Figure 2:**
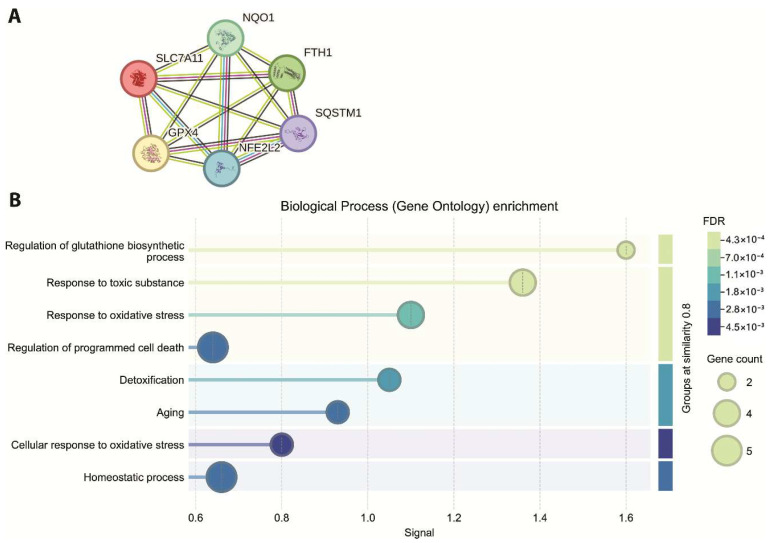
(**A**) PPI network of ferroptosis-related genes commonly altered across breast cancer molecular subtypes. (**B**) FDR profile of ferroptosis-related genes consistently differentially expressed across breast cancer subtypes.

Protein–protein interaction analysis was subsequently performed using the STRING database to assess connectivity among the six ferroptosis-related genes shared across all subtypes (Figure 2A). The resulting interaction network consisted of six nodes and 15 edges, corresponding to an average node degree of 5. The average local clustering coefficient was 1, indicating complete clustering among the nodes. The expected number of edges for a network of this size was 1, whereas the observed number of interactions was substantially higher. The calculated protein–protein interaction enrichment *p*-value was 1.11 × 10^−16^, confirming statistically significant interaction enrichment within the analyzed gene set. Functional enrichment analysis was conducted for the subset of ferroptosis-related genes that were identified as consistently differentially expressed across all breast cancer molecular subtypes. As shown in Figure 2B, multiple functional categories reached statistical significance after FDR correction. Enriched terms were ordered according to FDR values, and the number of genes contributing to each category ranged from two to five, as reflected by the relative marker sizes. The enrichment results demonstrated a non-random distribution of these genes across the identified functional categories.

### 2.7. Overall Survival of Breast Cancer Patients

**Figure 3:**
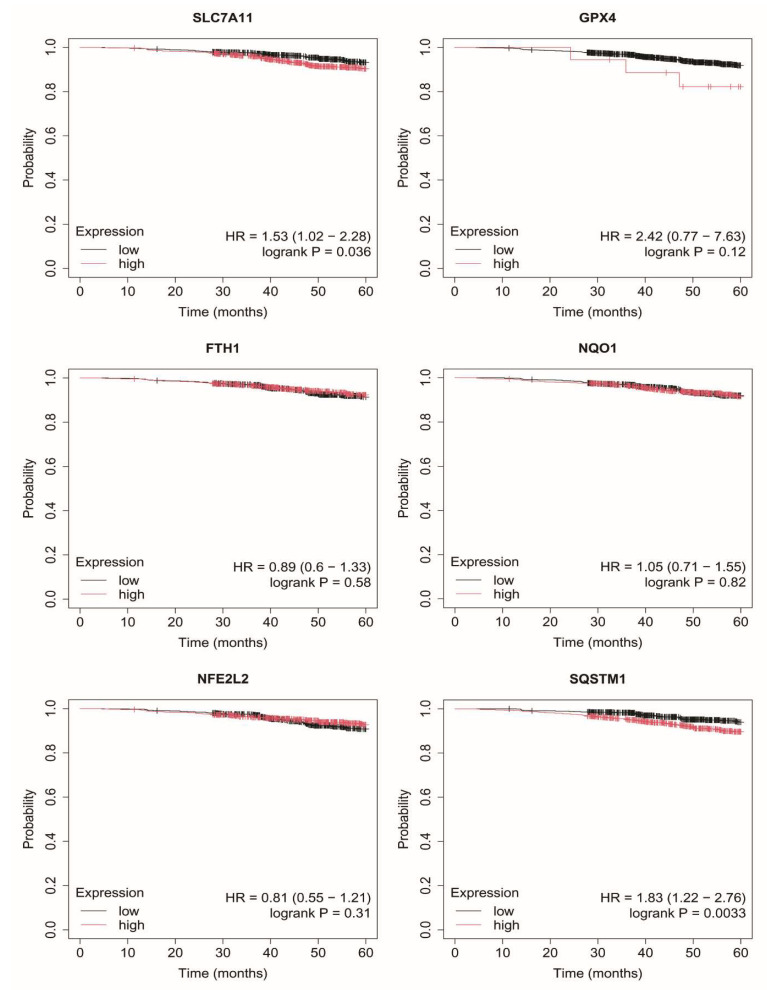
Overall survival analysis in luminal A cancer. SLC7A11, Solute carrier family 7 member 11 (cystine/glutamate antiporter, system Xc^−^ light chain); GPX4, Glutathione peroxidase 4; FTH1, Ferritin heavy chain 1; NQO1, NAD(P)H quinone dehydrogenase 1; NFE2L2, Nuclear factor erythroid 2-related factor 2; SQSTM1, Sequestosome 1.

**Figure 4:**
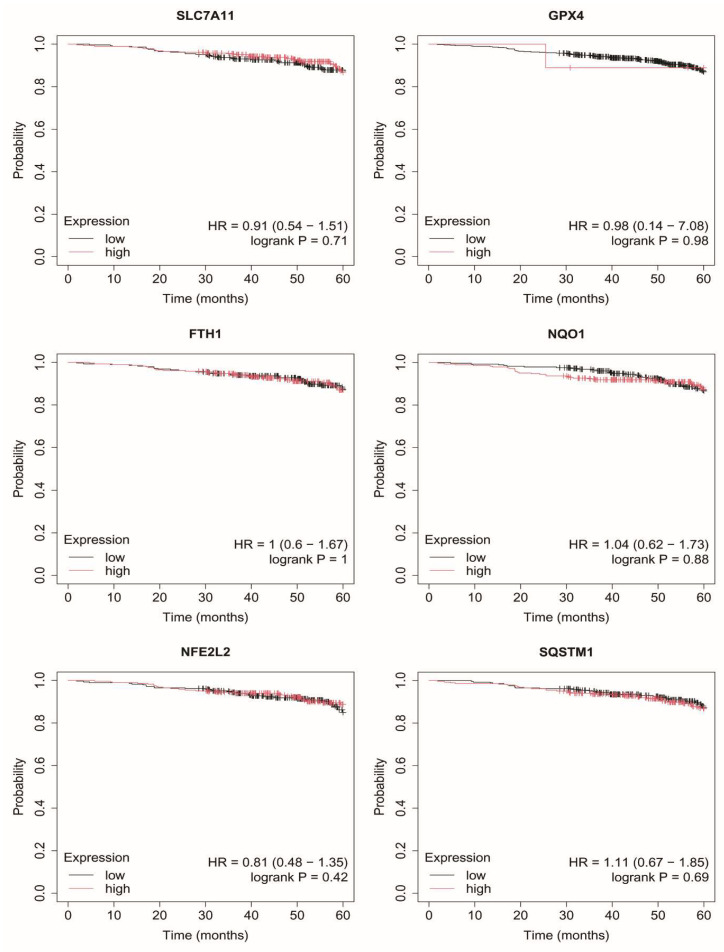
Overall survival analysis in luminal B HER2− cancer. SLC7A11, Solute carrier family 7 member 11 (cystine/glutamate antiporter, system Xc^−^ light chain); GPX4, Glutathione peroxidase 4; FTH1, Ferritin heavy chain 1; NQO1, NAD(P)H quinone dehydrogenase 1; NFE2L2, Nuclear factor erythroid 2-related factor 2; SQSTM1, Sequestosome 1.

**Figure 5:**
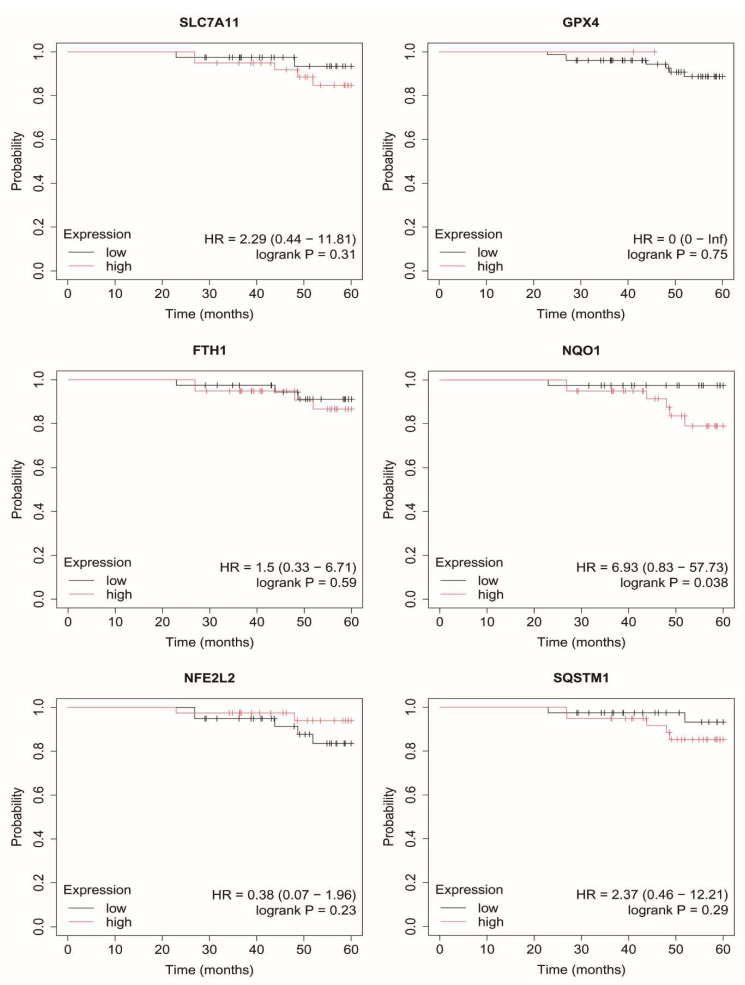
Overall survival analysis in luminal B HER2+ cancer. SLC7A11, Solute carrier family 7 member 11 (cystine/glutamate antiporter, system Xc^−^ light chain); GPX4, Glutathione peroxidase 4; FTH1, Ferritin heavy chain 1; NQO1, NAD(P)H quinone dehydrogenase 1; NFE2L2, Nuclear factor erythroid 2-related factor 2; SQSTM1, Sequestosome 1.

**Figure 6:**
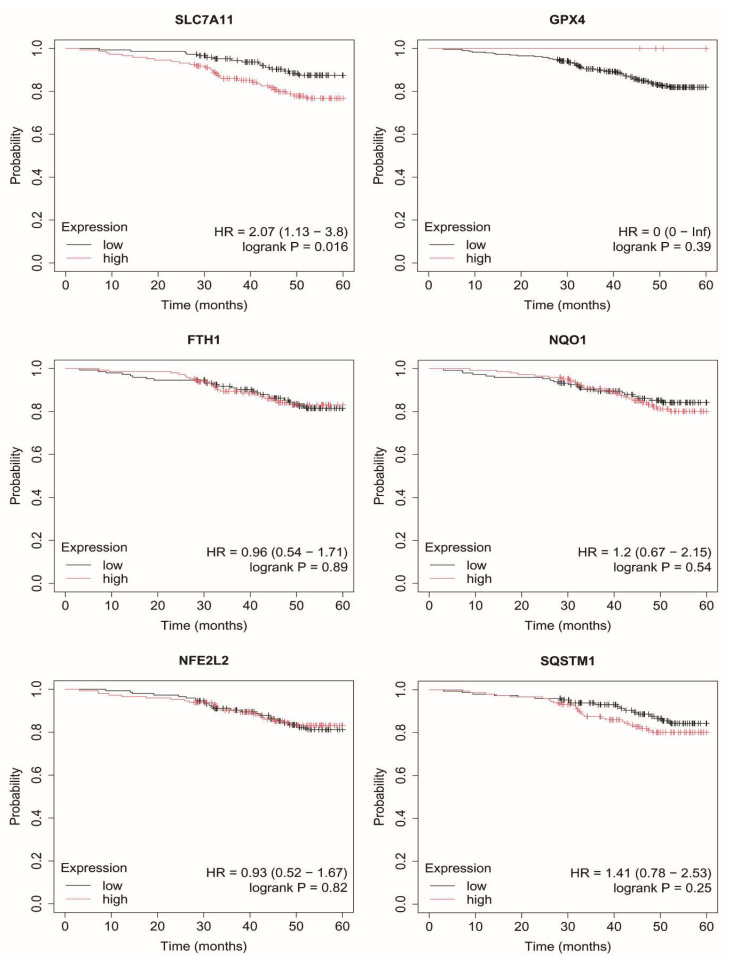
Overall survival analysis in non-luminal B HER2+ cancer. SLC7A11, Solute carrier family 7 member 11 (cystine/glutamate antiporter, system Xc^−^ light chain); GPX4, Glutathione peroxidase 4; FTH1, Ferritin heavy chain 1; NQO1, NAD(P)H quinone dehydrogenase 1; NFE2L2, Nuclear factor erythroid 2-related factor 2; SQSTM1, Sequestosome 1.

**Figure 7:**
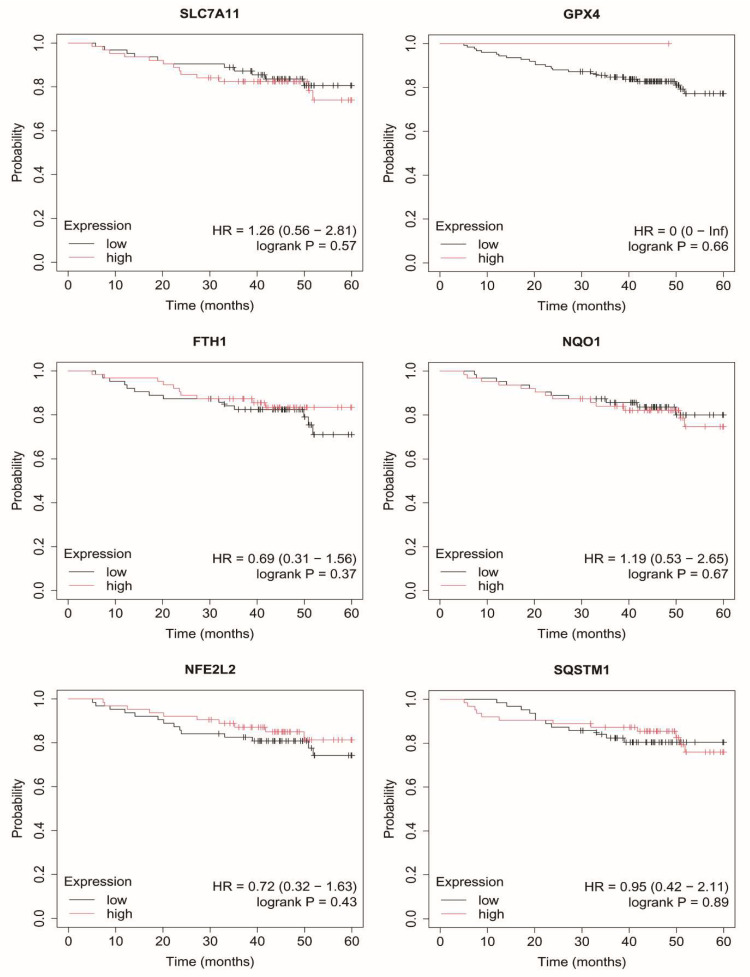
Overall survival analysis in TNBC. SLC7A11, Solute carrier family 7 member 11 (cystine/glutamate antiporter, system Xc^−^ light chain); GPX4, Glutathione peroxidase 4; FTH1, Ferritin heavy chain 1; NQO1, NAD(P)H quinone dehydrogenase 1; NFE2L2, Nuclear factor erythroid 2-related factor 2; SQSTM1, Sequestosome 1.

Exploratory overall survival analysis was performed for six mRNAs selected in the study (*SLC7A11*, *GPX4*, *FTH1*, *NQO1*, *NFE2L2*, *SQSTM1*) using the Kaplan–Meier Plotter online database (Figure 3, Figure 4, Figure 5, Figure 6 and Figure 7).

In luminal A breast cancer, higher expression levels of *SLC7A11* and *SQSTM1* were associated with poorer overall survival (Figure 3).

In contrast, in luminal B subtypes (HER2-negative and HER2-positive), no consistent or statistically robust associations between the expression of the analyzed genes and overall survival were observed (Figure 4 and Figure 5).

In non-luminal HER2-positive breast cancer, elevated *SLC7A11* expression was associated with poorer survival, whereas the remaining genes did not show significant associations (Figure 6).

In TNBC, none of the analyzed genes demonstrated a significant relationship with overall survival (Figure 7).

Overall, the observed associations were heterogeneous and subtype-dependent, with limited and gene-specific prognostic relevance. In addition, wide confidence intervals in certain subgroups, as well as instances of unstable estimates (e.g., hazard ratios approaching 0 or infinity), suggest limited robustness of some survival models, likely reflecting subgroup size constraints within the analyzed datasets. As these analyses were based on publicly available data, the findings should be interpreted as exploratory rather than as direct validation within the study cohort.

## 3. Discussion

The present study provides a comprehensive, multilevel characterization of ferroptosis-related molecular alterations in breast cancer, integrating transcriptomic, post-transcriptional, and protein-level data across five major molecular subtypes. By combining tumor tissue analyses with an independent in vivo model of controlled tissue injury induced by fibroadenoma cryoablation, this work offers novel insight into both local and systemic dimensions of ferroptosis-associated signaling in human breast pathology. Importantly, the present study is descriptive and integrative in nature and does not include experimental manipulation of ferroptosis pathways.

A central finding of this study is the identification of a subset of six ferroptosis-associated genes—*SLC7A11*, *GPX4*, *FTH1*, *NQO1*, *NFE2L2*, and *SQSTM1*—that were consistently upregulated across all breast cancer molecular subtypes at both the mRNA and protein levels. This subtype-independent expression pattern suggests that enhanced antioxidant capacity, iron handling, and redox homeostasis may represent fundamental adaptive features of breast cancer cells, irrespective of hormone receptor or HER2 status [36,37,38,39]. Functionally, these genes are known to participate in mechanisms that counteract lipid peroxidation and oxidative stress, the defining drivers of ferroptotic cell death [40]. Their coordinated upregulation is therefore consistent with a molecular profile compatible with ferroptosis resistance, although this interpretation remains indirect and requires functional validation [41,42].

A key observation is the consistent upregulation of SLC7A11 across all breast cancer subtypes. Elevated SLC7A11 expression is associated with enhanced cystine uptake and glutathione biosynthesis, which may contribute to the maintenance of redox balance and suppression of lipid peroxidation [43,44,45]. From a clinical perspective, this may indicate that breast cancers—particularly HER2-positive and TNBC tumors, where expression levels were highest—exhibit molecular features associated with reduced sensitivity to ferroptosis-inducing stress, including chemotherapy- or radiotherapy-associated oxidative damage [46]. SLC7A11 may therefore represent a potential target for ferroptosis sensitization strategies; however, its functional role in this context was not directly assessed in the present study [47,48].

Similarly, the observed upregulation of GPX4 at both mRNA and protein levels highlights its established role in detoxifying lipid hydroperoxides and limiting ferroptotic cell death [49]. The progressive increase in GPX4 expression across more aggressive subtypes may reflect enhanced antioxidant defense capacity [50]. This pattern may help explain reduced responsiveness to therapies that rely on oxidative stress-mediated cytotoxicity [51,52]. Although experimental studies have demonstrated that GPX4 inhibition can induce ferroptosis in cancer cells, the present findings should be interpreted as supportive rather than confirmatory of such mechanisms [52,53].

The increased expression of FTH1 (ferritin heavy chain) suggests enhanced intracellular iron sequestration, potentially limiting the availability of redox-active Fe^2+^ required for lipid peroxidation [54]. This may reflect an adaptive mechanism to buffer iron-mediated toxicity and reduce susceptibility to ferroptosis [55,56]. At the same time, elevated ferritin levels may serve as an indicator of altered iron homeostasis, which could be explored diagnostically or therapeutically, for example, in combination with strategies targeting iron metabolism [57,58].

Upregulation of NQO1 reflects reinforcement of antioxidant defense through quinone detoxification and protection against oxidative cycling. Increased NQO1 expression has been associated with chemoresistance and altered drug metabolism [59,60]. Its consistent elevation across subtypes suggests that ferroptosis-related pathways are closely linked to broader redox-regulatory mechanisms that may influence treatment response [61]. Given that NQO1 is a druggable enzyme, its expression may have potential relevance for patient stratification, although this requires further investigation [62,63].

The increased expression of NFE2L2 (NRF2) represents a central regulatory feature with potential clinical implications. NRF2 is known to orchestrate a coordinated antioxidant and cytoprotective transcriptional program [64], including regulation of SLC7A11, GPX4, FTH1, and NQO1. Sustained NRF2 activation has been associated with therapy resistance and tumor persistence [65,66,67]. The observed expression pattern may therefore be explained by activation of the NRF2 pathway, although this cannot be directly confirmed without functional assays [68,69].

The concurrent upregulation of SQSTM1 (p62) adds an additional layer of regulatory complexity. SQSTM1 participates in selective autophagy and can stabilize NRF2 through KEAP1 sequestration, thereby amplifying antioxidant signaling [70,71]. This suggests the presence of a potential regulatory loop integrating autophagy, redox control, and ferroptosis-related pathways [72]. From a translational perspective, this may indicate that combined targeting of autophagy and ferroptosis-associated pathways could be of interest, particularly in aggressive breast cancer subtypes, although further validation is required [73].

While these findings support the biological relevance of the identified genes, their direct impact on clinical outcomes appears to be context-dependent and was not consistently supported by survival analysis.

Beyond these shared alterations, the study revealed substantial subtype-specific heterogeneity among other ferroptosis-related genes. Differential expression of lipid metabolism enzymes (e.g., *ACLY*, *DHCR7*, *SC5D*), epigenetic regulators (*HDAC3*, *KAT2B*), and membrane transporters (*SLC25A1*, *SLC39A7*) suggests that ferroptosis susceptibility may be influenced by the broader metabolic and regulatory context of each molecular subtype [74,75,76]. For example, selective downregulation of cholesterol biosynthesis-related genes in luminal A and TNBC tumors may reflect differences in lipid remodeling that could affect membrane composition and vulnerability to lipid peroxidation [77,78]. These subtype-specific patterns may have implications for tailored ferroptosis-based therapeutic approaches [74,75,79].

The integrated miRNA analysis provides an additional post-transcriptional dimension. The observed downregulation of miRNAs predicted to target ferroptosis-related genes—such as miR-26a-5p targeting *SLC7A11* [80] or miR-28-5p targeting *NFE2L2* [81]—may partially contribute to the observed expression patterns; however, this mechanism should be interpreted within the context of dominant upstream transcriptional regulation.

A key feature of the molecular profile identified in this study is the coordinated expression of genes within the NRF2 signaling axis. *SLC7A11, GPX4, FTH1, NQO1, and SQSTM1* are known *NRF2* targets and are co-regulated as part of a cytoprotective response to oxidative stress [82,83]. *NRF2* activation promotes cystine uptake, glutathione synthesis, iron sequestration, and detoxification processes, collectively reducing susceptibility to ferroptosis [84,85]. The observed expression patterns are therefore consistent with NRF2 pathway activation, although this interpretation remains inferential and requires functional validation [86,87].

In this context, miRNA downregulation may act as a secondary modulatory mechanism reinforcing NRF2-driven gene expression rather than serving as a primary regulatory driver. This is consistent with a hierarchical regulatory model in which transcriptional activation predominates over post-transcriptional repression [88,89,90,91]. Such interactions may influence the balance between ferroptotic vulnerability and cellular survival, potentially affecting therapeutic responses [92,93].

An important aspect of the present study is the interpretation of survival analysis results, which were derived from the Kaplan–Meier Plotter database and therefore represent an external, exploratory assessment rather than a direct validation within the analyzed patient cohort. The observed associations were heterogeneous and subtype-dependent, with only selected genes—most notably *SLC7A11*—showing prognostic relevance in specific subgroups. In contrast, no consistent associations were observed in luminal B or TNBC subtypes.

These findings indicate that, although ferroptosis-related genes are consistently upregulated at the molecular level, their prognostic significance is limited and cannot be generalized across all breast cancer subtypes. The lack of association in TNBC, despite high expression levels, further suggests that these genes may reflect underlying tumor biology and oxidative stress adaptation rather than directly determining clinical outcomes. This distinction underscores the complexity of translating molecular alterations into clinically predictive biomarkers. These findings should be interpreted in the context of external, database-derived analyses rather than cohort-specific validation.

An additional strength of this study is the analysis of systemic molecular responses following fibroadenoma cryoablation, which provides an in vivo model of controlled tissue injury and regulated cell death [94]. The observed transient yet coordinated upregulation of ferroptosis-related mRNAs and proteins in peripheral blood partially recapitulates the molecular profile identified in breast cancer tissue. The clearly defined temporal pattern—early induction within hours after cryoablation followed by progressive normalization—supports the interpretation that these changes reflect an acute stress- and injury-related response, rather than long-term systemic reprogramming.

Importantly, these findings demonstrate that localized tissue damage associated with regulated cell death is sufficient to generate detectable circulating ferroptosis-related molecular signals [95]. This observation has direct practical implications, as it suggests that ferroptosis-associated molecules can be monitored in peripheral blood and may function as dynamic, minimally invasive indicators of tissue injury, treatment-induced cell death, or immune activation [34,96]. However, these observations should not be interpreted as direct evidence of ferroptosis occurring systemically.

Importantly, fibroadenoma represents a benign breast lesion with fundamentally different biological characteristics compared with malignant tumors. Therefore, the molecular changes observed following cryoablation should not be interpreted as direct surrogates of tumor-associated ferroptosis. Instead, this model provides a controlled in vivo context for studying systemic responses to acute tissue injury and oxidative stress. Consequently, extrapolation of these findings to breast cancer biology should be made with caution, and the observed systemic changes are best understood as reflecting general stress-response pathways that overlap with, but are not specific to, ferroptosis mechanisms active in malignant disease.

Taken together, the cryoablation data extend the relevance of tissue-based findings by demonstrating that ferroptosis-associated signaling is not confined to the local microenvironment but can be reflected systemically [97,98]. This opens potential avenues for the development of blood-based biomarkers to monitor therapeutic response, treatment-induced oxidative stress, or early biological effects of ferroptosis-modulating interventions in breast cancer and other solid tumors.

A critical methodological consideration concerns the interpretation of circulating protein levels measured following cryoablation. The analyzed proteins—SLC7A11, GPX4, FTH1, NQO1, NFE2L2, and SQSTM1—are well-characterized intracellular regulators of redox homeostasis and ferroptosis-related pathways; however, their utility as circulating biomarkers of ferroptosis remains insufficiently established. In the present study, these proteins were selected based on their robust and subtype-independent overexpression in breast cancer tissue and were subsequently assessed in serum to determine whether systemic changes could be detected following localized tissue injury. Therefore, the observed temporal increases in serum concentrations should be interpreted as reflecting a generalized systemic response to oxidative stress, inflammation, and cellular damage rather than direct evidence of circulating ferroptosis-specific biomarkers.

From a translational perspective, the results underscore both opportunities and challenges associated with targeting ferroptosis in breast cancer. While pharmacological induction of ferroptosis has shown promise in preclinical models, the observed upregulation of ferroptosis suppressors may indicate the presence of intrinsic resistance mechanisms in human tumors, particularly in aggressive subtypes [98,99]. Effective therapeutic strategies may therefore require combination approaches that simultaneously inhibit multiple ferroptosis-defense pathways or disrupt upstream regulators such as NRF2 signaling [76,87,99]. Moreover, the subtype-specific differences identified in ancillary pathways suggest that patient stratification will be essential for maximizing the efficacy of ferroptosis-based interventions [100].

Thus, while miRNA dysregulation may contribute to the stabilization of ferroptosis-resistant phenotypes, the coordinated expression pattern observed in this study is more consistent with activation of an upstream NRF2-dependent transcriptional program.

Several limitations of the present study should be acknowledged. First, although the cohort size was substantial and included all major breast cancer molecular subtypes, the analysis was restricted to early-stage (T1N0M0) tumors, which may limit extrapolation to advanced or metastatic disease. Second, the ferroptosis-related gene panel was derived from curated databases and does not fully capture the complexity of ferroptosis regulatory networks, including lipidomic alterations and metabolic fluxes that were not assessed in this study. Survival analyses were performed using publicly available datasets (Kaplan–Meier Plotter) rather than the study cohort and should therefore be interpreted as exploratory. The observed variability across molecular subtypes suggests that the prognostic relevance of ferroptosis-related genes may be context-dependent rather than uniform. Furthermore, the study is primarily based on correlative expression analyses and does not include direct functional validation of ferroptosis involvement. Similarly, the miRNA–mRNA interactions and protein–protein networks were inferred using established bioinformatic tools and should be interpreted as predictive.

In addition, the transcriptomic data utilized in this study were generated within a previously described experimental framework. However, the present work applies a distinct analytical approach focused specifically on ferroptosis-related pathways, providing new biological insights without duplication of experimental procedures.

Finally, while the cryoablation model offers valuable insight into systemic responses to localized tissue injury, fibroadenoma biology differs from malignant disease, and extrapolation of these findings to breast cancer should be made with caution.

Despite these limitations, the strength of the study lies in its integrative design combining transcriptomic, post-transcriptional, and protein-level analyses across multiple breast cancer subtypes. The consistency of the observed molecular patterns, particularly within NRF2-associated pathways, supports their biological relevance and provides a basis for further functional and clinical investigations.

## 4. Materials and Methods

This section incorporates selected methodological and background information that was previously described in our earlier study [101]. In the present work, these data were reanalyzed with a distinct biological focus on ferroptosis-related pathways using independent statistical and bioinformatic approaches. This strategy enables the extraction of novel biological insights without duplication of experimental procedures. The microarray experiment itself was performed once, in accordance with standard high-throughput transcriptomic protocols, and the current study represents a new analytical framework focused specifically on ferroptosis-related gene networks.

### 4.1. Study Design

The present investigation builds on our earlier research by extending the molecular scope to pathways implicated in ferroptosis in breast cancer [101,102,103,104]. The study was designed to provide a comprehensive characterization of ferroptosis-associated molecular alterations across the major molecular subtypes of breast cancer.

Breast tumor samples, together with paired histologically normal breast tissues, were obtained from patients representing the principal molecular subtypes of breast cancer, including luminal A, luminal B HER2-negative, luminal B HER2-positive, non-luminal HER2-positive, and triple-negative breast cancer (TNBC). Each tumor–control pair was subjected to a multistep molecular analysis. Initially, global transcriptomic profiling was performed using microarray platforms to assess the expression patterns of messenger RNA (mRNA) and microRNA (miRNA). Genes identified as significantly dysregulated were subsequently validated at the transcriptional level using qRT-PCR.

Based on the transcriptomic analysis, a subset of ferroptosis-related genes—*SLC7A11*, *GPX4*, *FTH1*, *NQO1*, *NFE2L2*, and *SQSTM1*—was selected for further investigation. These genes demonstrated consistent and statistically significant differential expression between breast cancer tissue and matched control samples across all molecular subtypes. The corresponding protein products were quantified using ELISA in both tumor and control tissue samples.

In the subsequent stage of the investigation, a separate comparative cohort consisting of patients with breast fibroadenoma undergoing cryoablation therapy was included. Peripheral blood and serum samples were collected at predefined time points, including immediately before the procedure and during post-treatment follow-up. Expression levels of the selected genes in peripheral blood were assessed using qRT-PCR, while circulating protein concentrations were measured using ELISA.

Within the fibroadenoma cohort, the analysis was restricted to the same panel of genes and proteins that had demonstrated reproducible, subtype-independent differential expression in breast cancer tissue. This approach enabled a comparative evaluation of local tumor-associated molecular alterations and systemic responses to controlled tissue injury.

Importantly, the assessment of these proteins in serum was exploratory in nature. Although these molecules are well-characterized intracellular regulators of ferroptosis-related pathways, their utility as circulating biomarkers of ferroptosis has not been definitively established. Therefore, changes in serum concentrations were interpreted cautiously as indirect indicators of systemic oxidative stress and injury-related responses rather than as specific markers of ferroptotic cell death.

### 4.2. Patients with Breast Cancer

The investigated cohort consisted of patients representing five molecular subtypes of breast cancer: luminal A (n = 130), luminal B HER2-negative (n = 100), luminal B HER2-positive (n = 96), non-luminal HER2-positive (n = 36), and triple-negative breast cancer (TNBC) (n = 43). During surgical procedures, both tumor tissue and a fragment of macroscopically unchanged breast tissue located at the surgical margin were collected. The latter served as the reference control material.

All tumors included in the study were classified as stage T1N0M0 according to the Tumor–Node–Metastasis (TNM) classification system [105].

Within the luminal A subgroup, histopathological grading revealed 23 tumors (18%) classified as G1, 48 cases (37%) as G2, and 59 tumors (45%) as G3. Regarding age distribution, 43 patients (33%) were younger than 50 years, whereas 87 individuals (67%) were aged 50 years or older. The average body mass index (BMI) in this subgroup was 30.78 ± 2.76 kg/m^2^.

In the luminal B HER2-negative group, tumor grading demonstrated 31 cases (31%) with G1 differentiation, 57 cases (57%) with G2, and 12 tumors (12%) classified as G3. Thirty-two patients (32%) were under 50 years of age, while 68 patients (68%) were older than 50 years. The mean BMI recorded in this subgroup was 30.18 ± 4.56 kg/m^2^.

Among individuals diagnosed with luminal B HER2-positive breast cancer, histological evaluation showed 23 tumors (24%) graded as G1, 57 tumors (59%) as G2, and 16 tumors (17%) as G3. Nineteen patients (20%) were younger than 50 years, whereas the remaining 77 patients (80%) were aged 50 years or above. The mean BMI for this group was 32.09 ± 6.19 kg/m^2^.

The non-luminal HER2-positive subgroup included 9 tumors (25%) graded as G1, 12 tumors (33%) as G2, and 15 tumors (42%) as G3. In this category, 9 patients (25%) were younger than 50 years, while 27 patients (75%) were aged at least 50 years. The average BMI for this subgroup was 33.18 ± 5.67 kg/m^2^.

In the TNBC subgroup, histological grading identified 14 tumors (32%) classified as G1, 21 tumors (49%) as G2, and 8 tumors (19%) as G3. Ten patients (23%) were younger than 50 years, whereas 33 patients (77%) were older than 50 years. The mean BMI recorded in this group was 34.67 ± 2.98 kg/m^2^.

During surgery, intraoperative histopathological examination was conducted to verify the absence of tumor cells at the resection margins using immunohistochemical techniques. When tumor infiltration at the margins was suspected, additional tissue was removed to ensure adequate oncological clearance. Following confirmation of clear margins, paired specimens consisting of tumor tissue and adjacent non-neoplastic breast tissue were secured and preserved for further molecular analyses.

### 4.3. Patients with Fibroadenoma

A total of 34 patients with histologically confirmed breast fibroadenoma who were scheduled for cryoablation therapy using the IceCure ProSense™ system (IceCure Medical HQ, Caesarea, Israel) were recruited in accordance with the manufacturer’s procedural guidelines. The mean age of the study participants was 35.87 ± 4.11 years, while the average body mass index (BMI) was 26.15 ± 4.98 kg/m^2^.

Peripheral venous blood and serum specimens were obtained at multiple predefined time points to monitor systemic molecular changes associated with the procedure. Sample collection was performed immediately before cryoablation (T0), followed by 30–60 min after treatment (T1), 8–12 h post-procedure (T2), 48–72 h after the intervention (T3), seven days later (T4), and during follow-up visits at one month (T5) and three months (T6).

### 4.4. Total RNA Extraction from Tissue

Total RNA was obtained from tissue samples using TRIzol reagent (Invitrogen, Carlsbad, CA, USA; Cat. No. 15596026) according to the supplier’s protocol. Isolation of RNA from whole blood specimens was performed with the PAXgene Blood RNA Kit (Qiagen, Valencia, CA, USA; Cat. No. 762174). The isolated RNA was subsequently subjected to an additional purification step using the RNeasy Mini Kit (QIAGEN, Hilden, Germany; Cat. No. 74104).

To remove potential contamination with genomic DNA, the RNA preparations were treated with DNase I (Fermentas International Inc., Burlington, ON, Canada; Cat. No. 18047019).

RNA quality was evaluated by electrophoretic separation on 1% agarose gels containing ethidium bromide (0.5 mg/mL), allowing visualization of the 28S and 18S ribosomal RNA bands. RNA concentration and purity were determined spectrophotometrically based on absorbance measurements at 260 nm.

### 4.5. Microarray Profiling of Ferroptosis-Related Genes

Genes involved in ferroptosis were selected through interrogation of the Molecular Signatures Database (MsigDB; accessed 10 November 2025). A search using the keyword “ferroptosis” identified 18 mRNA transcripts that fulfilled the predefined inclusion criteria.

The expression of these candidate genes in tumor tissues and matched control samples was evaluated using the HG-U133_A2 microarray platform (Affymetrix, Santa Clara, CA, USA) together with the GeneChip™ 3′ IVT PLUS Reagent Kit (Affymetrix; Cat. No. 902416). All experimental steps were conducted according to previously established procedures and in accordance with the manufacturer’s technical recommendations. Among the 22,277 probe sets represented on the array, 65 probes were annotated as targeting ferroptosis-associated genes.

The microarray workflow comprised synthesis of double-stranded complementary DNA (cDNA), amplification through in vitro transcription to generate amplified RNA (aRNA), enzymatic fragmentation of labeled transcripts, and subsequent hybridization to the array. Fluorescent signals were detected using an Affymetrix GeneArray Scanner 3000 7G, and raw image data were processed with GeneChip^®^ Command Console^®^ Software.(3000Dx v.2).

### 4.6. Global Profiling of Ferroptosis-Related miRNAs and Target Prediction

To explore potential microRNAs involved in the regulation of ferroptosis-related genes, miRNA expression profiling was performed using the GeneChip miRNA 2.0 Array (Affymetrix) in strict accordance with the manufacturer’s instructions. Differentially expressed miRNAs were identified by comparing profiles obtained from tumor specimens with those from matched control tissues.

Potential regulatory interactions between miRNAs and ferroptosis-associated mRNAs were predicted using two established bioinformatic resources: TargetScan (http://www.targetscan.org/, accessed on 20 November 2025) [106] and miRanda/mirDB (http://mirdb.org, accessed on 20 November 2025) [107]. These algorithms evaluate candidate interactions based on sequence complementarity, predicted binding energy, and evolutionary conservation of target sites [107,108]. Predicted interactions receiving scores greater than 80 were interpreted as highly reliable, whereas those with scores below 60 were considered with caution and included only when supported by additional evidence. Combining predictions derived from multiple databases increased the robustness of the inferred miRNA–mRNA regulatory networks associated with ferroptosis signaling pathways.

### 4.7. qRT-PCR Validation

Six genes selected on the basis of the microarray results were further examined using quantitative reverse transcription polymerase chain reaction (RT-qPCR). Amplification reactions were performed with the SensiFast SYBR No-ROX One-Step Kit (Bioline, London, UK) in accordance with the protocol recommended by the manufacturer.

Relative transcript levels were determined using the 2^−ΔΔCt^ method. Expression values obtained for control samples were set to a baseline fold change of 1, allowing comparison with tumor samples. Fold-change values exceeding 1 were interpreted as gene upregulation, whereas values lower than 1 indicated reduced expression. β-actin (*ACTB*) was used as the endogenous reference gene for normalization. The sequences of the primers used in the analysis are listed in Table 6.

### 4.8. Protein Quantification by ELISA

Protein expression levels of selected ferroptosis-related markers were quantified using commercially available ELISA kits in accordance with the manufacturers’ instructions. Analyses were conducted on breast tissue homogenates and on serum samples collected from patients with breast fibroadenoma.

The following ELISA kits were employed (all from MyBioSource, Inc., San Diego, CA, USA): Cystine/Glutamate Transporter ELISA Kit (SLC7A11; #MBS760866), Glutathione Peroxidase 4 (GPX4) ELISA Kit (#MBS2000338), Ferritin ELISA Kit (#MBS726976), NAD(P)H Dehydrogenase, Quinone 1 (NQO1) ELISA Kit (#MBS705270), NF-E2-related Factor 2 (NFE2L2) ELISA Kit (#MBS3803826), and Sequestosome 1 (SQSTM1) ELISA Kit (#MBS1608932).

Breast tissue samples were homogenized under cold conditions using appropriate lysis buffers supplemented with protease inhibitors. Homogenates were centrifuged to remove cellular debris, and the supernatants were collected for protein analysis. Serum samples from fibroadenoma patients were obtained by standard venipuncture, allowed to clot at room temperature, and centrifuged to separate the serum fraction. All samples were aliquoted and stored at −80 °C until analysis to avoid repeated freeze–thaw cycles.

Protein concentrations were determined by measuring absorbance using a microplate reader at wavelengths specified by each assay. Concentrations were calculated from standard curves generated for each ELISA kit and were expressed according to the manufacturers’ recommendations.

### 4.9. Statistical Analysis

All statistical analyses were performed using Statistica 13.0 PL software (StatSoft, Kraków, Poland) together with the Transcriptome Analysis Console (Affymetrix). The normality of data distribution was assessed using the Shapiro–Wilk test, with statistical significance set at *p* < 0.05. Depending on whether the variables followed a normal distribution, group comparisons were conducted using Student’s *t*-test or analysis of variance (ANOVA). When multiple comparisons were required, the Benjamini–Hochberg procedure was applied to control the false discovery rate, followed by Tukey’s post hoc test.

Survival analyses were carried out using the Kaplan–Meier Plotter online database (http://kmplot.com/, accessed on 20 November 2025) [109,110], which integrates gene expression and survival data from publicly available datasets. Importantly, these analyses were conducted independently of the patient cohort included in the present study and should be interpreted as external, exploratory validation rather than direct clinical correlation within the analyzed group. In addition, gene–gene and protein–protein interaction networks were explored using the STRING database (version 11.0; accessed 20 November 2025). The significance of network enrichment was evaluated using the log10(observed/expected) interaction ratio, while the false discovery rate (FDR) was adjusted according to the Benjamini–Hochberg correction method [111].

#### Sample Size Determination

The required sample size was estimated using an online sample size calculator [112] assuming a confidence level of 95%. Based on epidemiological data indicating that approximately 19,620 women were diagnosed with breast cancer in Poland in 2019 [113], the minimum number of participants necessary for adequate statistical power was determined to be 377.

To provide an epidemiological context for the analyzed cohort, the distribution of breast cancer molecular subtypes reported in national studies [113] was compared with the proportions observed in the present dataset. According to published data, the relative frequencies of molecular subtypes are as follows: luminal A (23.7%), luminal B HER2− (38.8%), luminal B HER2+ (14%), non-luminal HER2+ (11.2%), and triple-negative breast cancer (TNBC) (12.3%). These values correspond closely with the subtype distribution identified in the current study population.

## 5. Conclusions

This study shows that breast cancer is characterized by coordinated activation of ferroptosis-associated regulatory pathways at the transcriptional, post-transcriptional, and protein levels across multiple molecular subtypes. The consistent upregulation of SLC7A11, GPX4, FTH1, NQO1, NFE2L2, and SQSTM1 suggests a shared molecular response related to antioxidant defense, iron handling, and redox homeostasis, although its clinical implications appear to be context-dependent.

The integrated miRNA analysis indicates that reduced post-transcriptional regulation may contribute to the sustained expression of key ferroptosis-associated genes; however, these patterns are most consistently explained by activation of NRF2-dependent transcriptional programs. Together, these findings are consistent with the concept that ferroptosis resistance in breast cancer may be associated with multilayered regulatory networks involving iron metabolism, glutathione-dependent detoxification, and oxidative stress response pathways.

Importantly, the cryoablation model demonstrates that ferroptosis-associated molecular signals can be detected systemically following localized tissue injury. However, these changes should be interpreted as reflecting general stress-response mechanisms rather than specific markers of ferroptosis.

Overall, the results highlight the biological relevance of ferroptosis-associated pathways in breast cancer while indicating that their prognostic significance is limited and subtype-dependent. These findings provide a foundation for further investigation into the role of ferroptosis in tumor biology and its potential exploitation in therapeutic strategies and biomarker development. Future studies integrating functional validation, treatment-response data, and longitudinal clinical outcomes will be necessary to define their translational value.

## Figures and Tables

**Table 1:** Differential expression of ferroptosis-related mRNAs across breast cancer molecular subtypes (mean ± SD).

Probe Set ID	mRNA	Luminal A vs. Control	Luminal B HER2− vs. Control	Luminal B HER2+ vs. Control	Non-Luminal HER2+ vs. Control	TNBC vs. Control
207528_s_at	*SLC7A11*	+3.42 *	+3.80 *	+4.13 *	+4.43 *	+4.76 *
209921_at		+3.22 *	+3.63 *	+3.93 *	+4.12 *	+4.53 *
217678_at		+3.34 *	+3.72 *	+4.04 *	+4.34 *	+4.62 *
201106_at	*GPX4*	+3.18 *	+3.41 *	+3.65 *	+3.91 *	+4.21 *
200748_s_at	*FTH1*	+3.21 *	+3.52 *	+3.78 *	+4.04 *	+4.39 *
200748_x_at		+3.19 *	+3.43 *	+3.64 *	+3.95 *	+4.14 *
201467_s_at	*NQO1*	+3.32 *	+3.65 *	+3.91 *	+4.22 *	+4.51 *
201468_s_at		+3.24 *	+3.52 *	+3.83 *	+4.11 *	+4.44 *
210519_s_at		+3.46 *	+3.74 *	+4.03 *	+4.34 *	+4.66 *
201146_at	*NFE2L2*	+3.02 *	+3.35 *	+3.63 *	+3.91 *	+4.11 *
1567013_at		+2.94 *	+3.28 *	+3.55 *	+3.87 *	+4.04 *
1567015_at		+3.17 *	+3.41 *	+3.77 *	+4.04 *	+4.28 *
201471_s_at	*SQSTM1*	+3.21 *	+3.56 *	+3.71 *	+4.14 *	+4.43 *
213112_s_at		+3.17 *	+3.43 *	+3.68 *	+4.06 *	+4.32 *
235530_at		+3.32 *	+3.62 *	+3.82 *	+4.22 *	+4.52 *
239004_at		+3.02 *	+3.36 *	+3.65 *	+3.96 *	+4.17 *
242568_s_at		+3.14 *	+3.43 *	+3.74 *	+4.03 *	+4.28 *
244804_at		+3.21 *	+3.56 *	+3.83 *	+4.12 *	+4.33 *
203665_at	*HMOX1*	+1.98 (ns)	+3.22 *	+3.52 *	+3.85 *	+4.02 *
224461_s_at	*AIFM2 (FSP1)*	+1.63 (ns)	+2.11 (ns)	+3.38 *	+3.65 *	+3.92 *
228445_at		+1.7 2(ns)	+2.05 (ns)	+3.21 *	+3.52 *	+3.88 *
201790_s_at	*DHCR7*	−3.12 *	−1.64 (ns)	−1.81 (ns)	−1.52 (ns)	−3.64 *
201791_s_at		−3.01 *	−1.55 (ns)	−1.75 (ns)	−1.46 (ns)	−3.52 *
211423_s_at	*SC5D*	−3.33 *	−1.71 (ns)	−1.93 (ns)	−1.67 (ns)	−3.82 *
215064_at		−3.14 *	−1.65 (ns)	−1.83 (ns)	−1.52 (ns)	−3.68 *
201127_s_at	*ACLY*	+2.01 (ns)	+3.11 *	+2.21 (ns)	+2.09 (ns)	+3.72 *
201128_s_at		+2.11 (ns)	+3.03 *	+2.35 (ns)	+2.12 (ns)	+3.61 *
210337_s_at		+1.91 (ns)	+3.26 *	+2.15 (ns)	+2.04 (ns)	+3.88 *
210010_s_at	*SLC25A1*	+1.85 (ns)	+3.03 *	+2.14 (ns)	+1.95 (ns)	+3.53 *
216326_s_at	*HDAC3*	+1.55 (ns)	+3.02 *	+1.70 (ns)	+1.61 (ns)	+1.93 (ns)
203845_at	*KAT2B*	−3.04 *	−1.54 (ns)	−1.44 (ns)	−1.64 (ns)	−1.84 (ns)
220486_x_at	*TMEM164*	+1.72 (ns)	+1.94 (ns)	+3.14 *	+2.16 (ns)	+2.02 (ns)
223201_s_at		+1.62 (ns)	+1.85 (ns)	+3.02 *	+2.02 (ns)	+1.92 (ns)
223202_s_at		+1.83 (ns)	+2.09 (ns)	+3.22 *	+2.25 (ns)	+2.16 (ns)
202667_s_at	*SLC39A7*	+1.64 (ns)	+1.84 (ns)	+3.24 *	+2.06 (ns)	+2.10 (ns)
206582_s_at	*ADGRG1*	+1.43 (ns)	+1.67 (ns)	+1.82 (ns)	+3.12 *	+1.93 (ns)
212070_at		+1.51 (ns)	+1.75 (ns)	+1.96 (ns)	+3.22 *	+2.01 (ns)
203045_at	*NINJ1*	+1.52 (ns)	+1.73 (ns)	+1.91 (ns)	+3.37 *	+2.06 (ns)

Values represent log_2_ fold change in mRNA expression between breast cancer tissue and matched non-neoplastic control samples as determined by microarray analysis. An asterisk (*) denotes statistically significant differential expression (*p* < 0.05) based on analysis of variance followed by Tukey’s post hoc test, whereas “ns” indicates a change that did not reach statistical significance. Multiple probe sets corresponding to the same gene are shown to reflect probe-level representation and consistency on the Affymetrix HG-U133_A2 microarray platform. *ACLY*, ATP citrate lyase; *ADGRG1*, Adhesion G protein-coupled receptor G1; *AIFM2 (FSP1)*, Apoptosis-inducing factor mitochondria-associated 2 (ferroptosis suppressor protein 1); *DHCR7*, 7-dehydrocholesterol reductase; *FTH1*, Ferritin heavy chain 1; *GPX4*, Glutathione peroxidase 4; *HDAC3*, Histone deacetylase 3; *HMOX1*, Heme oxygenase 1; *KAT2B*, Lysine acetyltransferase 2B; *NFE2L2*, Nuclear factor erythroid 2-related factor 2; *NINJ1*, Ninjurin 1; *NQO1*, NAD(P)H quinone dehydrogenase 1; *SC5D*, Sterol-C5-desaturase; *SLC7A11*, Solute carrier family 7 member 11 (cystine/glutamate antiporter, system Xc^−^ light chain); *SLC25A1*, Solute carrier family 25 member 1 (mitochondrial citrate transporter); *SLC39A7*, Solute carrier family 39 member 7 (zinc transporter ZIP7); *SQSTM1*, Sequestosome 1; *TMEM164*, Transmembrane protein 164; HER2, human epidermal growth factor receptor 2; TNBC, triple-negative breast cancer.

**Table 2:** Expression patterns of miRNAs predicted to regulate selected mRNAs in breast cancer subtype tissues relative to control samples (mean ± SD).

mRNA	miRNA	Target Score	Luminal A vs. Control (log_2_FC)	Luminal B HER2− vs. Control (log_2_FC)	Luminal B HER2+ vs. Control (log_2_FC)	Non-luminal Her2+ vs. Control (log_2_FC)	TNBC vs. Control (log_2_FC)
*SLC7A11*	hsa-miR-1297	100	−1.69 ± 0.13 *	−1.89 ± 0.43 *	−1.34 ± 0.12 *	−1.19 ± 0.22 *	−1.78 ± 0.19 *
	hsa-miR-26a-5p	100	−2.34 ± 0.12 *	−2.78 ± 0.76 *	−3.33 ± 0.34 *	−3.51 ± 0.21 *	−3.67 ± 0.26 *
*NQO1*	hsa-miR-18b-3p	82	−2.11 ± 0.81 *	−2.34 ± 0.18 *	−2.34 ± 019 *	−3.87 ± 0.19 *	−3.45 ± 0.55 *
*NFE2L2*	hsa-miR-28-5p	82	−3.23 ± 0.87 *	−3.21 ± 0.13 *	−2.34 ± 0.65 *	−3.87 ± 0.17 *	−3.81 ± 0.41 *

*SLC7A11*, Solute carrier family 7 member 11 (cystine/glutamate antiporter, system Xc^−^ light chain); *NQO1*, NAD(P)H quinone dehydrogenase 1; *NFE2L2*, Nuclear factor erythroid 2-related factor 2; HER2, human epidermal growth factor receptor 2; TNBC, triple-negative breast cancer; *, statistically significant differences compared with control samples (*p* < 0.05).

**Table 3:** Quantitative assessment of ferroptosis-associated protein levels in breast cancer and control tissues measured by ELISA (mean ± SD).

Protein	Control Tissue	Luminal A	Luminal B HER2−	Luminal B HER2+	Non-Luminal HER2+	TNBC
SLC7A11(ng/mL)	0.84 ± 0.12	2.31 ± 0.38 *	2.78 ± 0.44 *	3.35 ± 0.51 *	3.92 ± 0.63 *	4.48 ± 0.71 *
GPX4 (ng/mL)	28.6 ± 4.9	61.40 ± 9.70 *	72.3 ± 11.21 *	86.72 ± 13.40 *	102.93± 15.62 *	118.4 ± 18.1 *
FTH1 (ng/mL)	214.87 ± 36.12	398.98 ± 64.53 *	472.12 ± 71.98 *	556.09 ± 83.76 *	648.12 ± 97.09 *	742.12 ± 11.22 *
NQO1 (pg/mL)	182.91 ± 29.12	396.01 ± 61.98 *	468.54 ± 73.91 *	554.12 ± 86.91 *	639.11 ± 94.90 *	731.91 ± 108.12 *
NFE2L2(ng/mL) *	12.8 ± 2.10	24.62 ± 3.90 *	29.30 ± 4.71 *	34.81 ± 5.63 *	40.94 ± 6.34 *	46.70 ± 7.25 *
SQSTM1(ng/mL)	2.14 ± 0.41	4.83 ± 0.79 *	5.71 ± 0.92 *	6.74 ± 1.05 *	7.92 ± 1.21 *	9.18 ± 1.43 *

SLC7A11, Solute carrier family 7 member 11 (cystine/glutamate antiporter, system Xc^−^ light chain); GPX4, Glutathione peroxidase 4; FTH1, Ferritin heavy chain 1; NQO1, NAD(P)H quinone dehydrogenase 1; NFE2L2, Nuclear factor erythroid 2-related factor 2; SQSTM1, Sequestosome 1; HER2, human epidermal growth factor receptor 2; TNBC, triple-negative breast cancer; *, statistically significant differences compared with control samples (*p* < 0.05).

**Table 4:** Temporal Changes in mRNA Expression of Selected Genes in Women Diagnosed with Fibroadenoma (mean ± SD).

mRNA	T1 vs. T0 (FC)	T2 vs. T0 (FC)	T3 vs. T0 (FC)	T4 vs. T0 (FC)	T5 vs. T0 (FC)	T6 vs. T0 (FC)
*SLC7A11*	1.30 ± 0.18	1.92 ± 0.26 *	1.54 ± 0.22 *	1.14 ± 0.16	1.04 ± 0.14	1.01 ± 0.12
*GPX4*	1.24 ± 0.17	1.75 ± 0.24 *	1.46 ± 0.21 *	1.12 ± 0.15	1.03 ± 0.13	1.01 ± 0.11
*FTH1*	1.36 ± 0.20	2.03 ± 0.29 *	1.63 ± 0.24 *	1.20 ± 0.17	1.06 ± 0.15	1.02 ± 0.13
*NQO1*	1.29 ± 0.18	1.85 ± 0.25 *	1.52 ± 0.22 *	1.16 ± 0.16	1.04 ± 0.14	1.01 ± 0.12
*NFE2L2*	1.22 ± 0.16	1.66 ± 0.23 *	1.39 ± 0.20 *	1.10 ± 0.15	1.03 ± 0.13	1.01 ± 0.11
*SQSTM1*	1.34 ± 0.19	1.98 ± 0.28 *	1.59 ± 0.23 *	1.18 ± 0.17	1.05 ± 0.14	1.02 ± 0.12

*SLC7A11*, Solute carrier family 7 member 11 (cystine/glutamate antiporter, system Xc^−^ light chain); *GPX4*, Glutathione peroxidase 4; *FTH1*, Ferritin heavy chain 1; *NQO1*, NAD(P)H quinone dehydrogenase 1; *NFE2L2*, Nuclear factor erythroid 2-related factor 2; *SQSTM1*, Sequestosome 1; FC, fold-change. *, statistically significant differences compared with control samples (*p* < 0.05).

**Table 5:** Time-Course of Protein Concentrations for Selected Gene Products in Women with Fibroadenoma Treated with Cryoablation (mean ± SD).

Protein	T0 (Baseline)	T1 (30–60 min)	T2 (8–12 h)	T3 (48–72 h)	T4 (7 Days)	T5 (1 Month)	T6 (3 Months)
SLC7A11 (ng/mL)	0.92 ± 0.14	1.24 ± 0.19 *	1.86 ± 0.27 *	1.43 ± 0.22 *	1.05 ± 0.16	0.96 ± 0.14	0.93 ± 0.13
GPX4 (ng/mL)	32.4 ± 5.1	41.7 ± 6.3 *	58.9 ± 8.7 *	47.2 ± 7.1 *	36.9 ± 5.6 *	33.8 ± 5.2	32.9 ± 5.0
FTH1 (ng/mL)	238 ± 39	312 ± 48 *	426 ± 67 *	351 ± 55 *	271 ± 42 *	246 ± 38	240 ± 37
NQO1 (pg/mL)	196 ± 31	268 ± 43 *	382 ± 61 *	309 ± 49 *	231 ± 36 *	204 ± 32	198 ± 30
NFE2L2 (ng/mL)	14.2 ± 2.3	18.9 ± 3.0 *	26.4 ± 4.1 *	21.7 ± 3.5 *	16.3 ± 2.6 *	14.8 ± 2.4	14.4 ± 2.3
SQSTM1 (ng/mL)	2.36 ± 0.44	3.12 ± 0.56 *	4.48 ± 0.79 *	3.71 ± 0.66 *	2.78 ± 0.51 *	2.44 ± 0.46	2.39 ± 0.45

SLC7A11, Solute carrier family 7 member 11 (cystine/glutamate antiporter, system Xc^−^ light chain); GPX4, Glutathione peroxidase 4; FTH1, Ferritin heavy chain 1; NQO1, NAD(P)H quinone dehydrogenase 1; NFE2L2, Nuclear factor erythroid 2-related factor 2; SQSTM1, Sequestosome 1. *, statistically significant differences compared with control samples (*p* < 0.05).

**Table 6:** Nucleotide primers sequence.

mRNA	Sequence (5′-3′)
*SLC7A11*	Forward: TCCGATCTTTGTTGCCCTCT Reverse: GACTGTCGAGGTCTCCAGAG
*GPX4*	Forward: GCCAGGGAGTAACGAAGAGA Reverse: CAGCCGTTCTTGTCGATGAG
*FTH1*	Forward: ACTTTGACCGCGATGATGTG Reverse: GCTCTCCCAGTCATCACAGT
*NQO1*	Forward: TCCCAGGTTCCAGCAATTCT Reverse: CACTTTGGGAGGCTGAGGTA
*NFE2L2*	Forward: GGTTGCCCACATTCCCAAAT Reverse: AGCAATGAAGACTGGGCTCT
*SQSTM1*	Forward: AGGACAAATTGCGCCCATTT Reverse: TCTCTTTCAGGGACAGGCTG
*ACTB*	Forward: TCACCCACACTGTGCCCATCTACGA Reverse: CAGCGGAACCGCTCATTGCCAATGG

*SLC7A11*, Solute carrier family 7 member 11 (cystine/glutamate antiporter, system Xc^−^ light chain); *GPX4*, Glutathione peroxidase 4; *FTH1*, Ferritin heavy chain 1; *NQO1*, NAD(P)H quinone dehydrogenase 1; *NFE2L2*, Nuclear factor erythroid 2-related factor 2; *SQSTM1*, Sequestosome 1; *ACTB*, β-actin.

## Data Availability

The original contributions presented in this study are included in the article. Further inquiries can be directed to the corresponding authors.

## Abbreviations

ACLY	ATP citrate lyase
ADGRG1	Adhesion G protein-coupled receptor G1
AIFM2 (FSP1)	Apoptosis-inducing factor mitochondria-associated 2 (ferroptosis suppressor protein 1)
ANOVA	Analysis of variance
BMI	Body mass index
cDNA	Complementary DNA
Ct	Cycle threshold
ELISA	Enzyme-linked immunosorbent assay
FDR	False discovery rate
FC	Fold change
FTH1	Ferritin heavy chain 1
GPX4	Glutathione peroxidase 4
HDAC3	Histone deacetylase 3
HER2	Human epidermal growth factor receptor 2
HMOX1	Heme oxygenase 1
IHC	Immunohistochemistry
miRNA	MicroRNA
mRNA	Messenger RNA
MSigDB	Molecular Signatures Database
NFE2L2 (NRF2)	Nuclear factor erythroid 2-related factor 2
NINJ1	Ninjurin 1
NQO1	NAD(P)H quinone dehydrogenase 1
PPI	Protein–protein interaction
qRT-PCR	Quantitative reverse transcription polymerase chain reaction
RNA	Ribonucleic acid
RT	Reverse transcription
SC5D	Sterol-C5-desaturase
SD	Standard deviation
SLC25A1	Solute carrier family 25 member 1
SLC39A7	Solute carrier family 39 member 7
SLC7A11	Solute carrier family 7 member 11 (cystine/glutamate antiporter, system Xc^−^ light chain)
SQSTM1	Sequestosome 1
STRING	Search Tool for the Retrieval of Interacting Genes/Proteins
TNBC	Triple-negative breast cancer
TMEM164	Transmembrane protein 164
TNM	Tumor–Node–Metastasis classification
